# Oridonin stabilizes retinoic acid receptor alpha through ROS-activated NF-κB signaling

**DOI:** 10.1186/s12885-015-1219-8

**Published:** 2015-04-10

**Authors:** Yang Cao, Wei Wei, Nan Zhang, Qing Yu, Wen-Bin Xu, Wen-Jun Yu, Guo-Qiang Chen, Ying-Li Wu, Hua Yan

**Affiliations:** 1 Department of Hematology, Rui-Jin Hospital, Shanghai Jiao-Tong University School of Medicine, Shanghai, China; 2 Department of Hematology, Xinhua Hospital, Shanghai Jiao-Tong University School of Medicine, Shanghai, China; 3 Department of Pathophysiology, Chemical Biology Division of Shanghai Universities E-Institutes, Key Laboratory of Cell Differentiation and Apoptosis of National Ministry of Education, Shanghai Jiao-Tong University School of Medicine, Shanghai, China

**Keywords:** RARα, Oridonin, ROS, NF-κB

## Abstract

**Background:**

Retinoic acid receptor alpha (RARα) plays an essential role in the regulation of many biological processes, such as hematopoietic cell differentiation, while abnormal RARα function contributes to the pathogenesis of certain diseases including cancers, especially acute promyelocytic leukemia (APL). Recently, oridonin, a natural diterpenoid isolated from *Rabdosia rubescens*, was demonstrated to regulate RARα by increasing its protein level. However, the underlying molecular mechanism for this action has not been fully elucidated.

**Methods:**

In the APL cell line, NB4, the effect of oridonin on RARα protein was analyzed by western blot and real-time quantitative RT-PCR analyses. Flow cytometry was performed to detect intracellular levels of reactive oxygen species (ROS). The association between nuclear factor-kappa B (NF-κB) signaling and the effect of oridonin was assessed using specific inhibitors, shRNA gene knockdown, and immunofluorescence assays. In addition, primary leukemia cells were treated with oridonin and analyzed by western blot in this study.

**Results:**

RARα possesses transcriptional activity in the presence of its ligand, all-trans retinoic acid (ATRA). Oridonin remarkably stabilized the RARα protein, which retained transcriptional activity. Oridonin also moderately increased intracellular ROS levels, while pretreatment with the ROS scavenger, *N*-acetyl-l-cysteine (NAC), dramatically abrogated RARα stabilization by oridonin. More intriguingly, direct exposure to low concentrations of H_2_O_2_ also increased RARα protein but not mRNA levels, suggesting a role for ROS in oridonin stabilization of RARα protein. Further investigations showed that NAC antagonized oridonin-induced activation of NF-κB signaling, while the NF-κB signaling inhibitor, Bay 11–7082, effectively blocked the oridonin increase in RARα protein levels. In line with this, over-expression of IκΒα (A32/36), a super-repressor form of IκΒα, or NF-κB-p65 knockdown inhibited oridonin or H_2_O_2_-induced RARα stability. Finally, tumor necrosis factor alpha (TNFα), a classical activator of NF-κB signaling, modulated the stability of RARα protein.

**Conclusions:**

Oridonin stabilizes RARα protein by increasing cellular ROS levels, which causes activation of the NF-κB signaling pathway.

## Background

Retinoid receptors are retinoid ligand-activated transcription factors that are divided into retinoic acid receptors (RARs) and retinoid X receptors (RXRs). Both RARs and RXRs have three isoforms, including RARα/β/γ and RXRα/β/γ. These proteins are encoded by distinct loci and exist as alternatively spliced variants [1]. Active retinoid receptors consist of RAR/RXR heterodimers, which activate the transcription of many target genes by binding retinoic acid responsive elements in promoter and/or enhancer regions. They exert many essential and potent biological functions with respect to the regulation of cell proliferation, differentiation, apoptosis, and autophagy [2-4]. Accordingly, retinoids and their receptors are also widely involved in the pathogenesis of many diseases, especially cancers [5]. A typical example is that of acute promyelocytic leukemia (APL), a unique subtype of acute myeloid leukemia (AML). Almost all APL patients carry chromosome translocations involving *RAR*α*,* most of which are t(15;17). This causes fusion of the *promyelocytic leukemia* (*PML*) gene to the *RAR*α gene and expression of a *PML-RAR*α fusion gene, leading to impaired retinoid signaling and pathogenesis of APL. Importantly, all-trans retinoic acid (ATRA) and arsenic trioxide target the PML-RARα fusion protein to induce differentiation and/or apoptosis of leukemia-initiating cells [6-10]. Besides APL, some other types of cancer also present with aberrant expression of RARs. For example, the expression of RARα/β and RXRα/β are down-regulated in pancreatic ductal adenocarcinoma, which is associated with poor patient survival outcomes [11].

The mechanisms regulating the expression of RARs are not fully understood. ATRA can directly target RARα to ubiquitin-proteasome degradation in APL and non-APL cells [12], while activation of c-Jun N-terminal kinase (JNK) can contribute to RAR dysfunction by phosphorylating RARα at Thr181, Ser445, and Ser461. This induces RAR degradation through the ubiquitin-proteasome pathway, pointing to JNK as a key mediator of aberrant retinoid signaling in lung cancer cells [13]. Additionally, JNK activation by oxidative stress also suppresses retinoid signaling through proteasomal degradation of RARα in hepatic cells [14]. More recently, pharicin B, a novel natural *ent-kaurene* diterpenoid derived from *Isodon pharicus* leaves, was reported to rapidly stabilize RARα protein in various AML cell lines and primary leukemic cells from AML patients [15].

Oridonin, another *ent-kaurene* diterpenoid isolated from *Rabdosia rubescens*, has a variety of biological effects, such as anti-inflammatory, anti-viral, and anti-bacterial functions, as well as anti-tumor effects on different cancers including liver [16], prostate [17], breast [18], and leukemia [19]. Accumulating evidence illustrates that oridonin has extensive anti-tumor effects involving regulation of the cell cycle, apoptosis, autophagy, and differentiation [20-22]. Previously, we reported that oridonin could induce ROS-initiated apoptosis and enhance ATRA-induced differentiation in APL cells. Interestingly, the differentiation-enhancing effect of oridonin was accompanied by increased levels of RARα protein [23]. In this work, we further investigated the mechanisms underlying oridonin stabilization of RARα protein.

## Methods

### Cells

NB4/GFP and NB4/GFP-MAD cells were generous gifts from F. Besancon (Hôpital St. Louis, Paris, France). Construction of the two cell lines was described previously by Komura et al. [24]. NB4, NB4/GFP, and NB4/GFP-MAD cells were cultured in RPMI 1640 medium (Sigma-Aldrich, St. Louis, MO, USA), supplemented with 10% (v/v) heat-inactivated fetal bovine serum (FBS; HyClone, Logan, UT, USA). COS-7 and 293 T cells were cultured in Dulbecco’s modified Eagle’s medium (Life Technologies, USA), supplemented with 10% FBS in a humidified incubator at 37°C with 5% CO_2_/95% air (v/v).

### Reagents and antibodies

Oridonin (purity >99.5%) was purchased from Xi’an Haoxuan Biotechnique, China. It was dissolved in dimethyl sulfoxide (DMSO) at a stock concentration of 10 mM and stored at −20°C. Both *N*-acetyl-l-cysteine (NAC) and ATRA were purchased from Sigma-Aldrich. Recombinant human tumor necrosis factor (TNFα) was obtained from Peprotech (Rocky Hill, NJ, USA). Cycloheximide was purchased from Sigma-Aldrich. ERK inhibitor PD98059, p38 inhibitor SB203580, JNK inhibitor SP600125, and NF-κB inhibitor Bay 11–7082 were purchased from Santa Cruz Biotechnology (Santa Cruz, CA, USA). When cells were treated with these reagents, matching concentrations of vehicle were used as the control and the final concentration of DMSO was kept at or below 0.1% in all experiments.

Antibodies recognizing p65, IκBα, and RARα were purchased from Santa Cruz Biotechnology. Antibodies recognizing phospho-IκBα (Ser32/Ser36), phospho-p65, IκB kinase beta (IKKβ), phospho-IKKα/β, phospho-ERK1/ERK2, ERK1/ERK2, phospho-p38, p38, phospho-JNK, and JNK were purchased from Cell Signaling Technology (Beverly, MA, USA).

### Western blot

Equal amounts of protein extracts were loaded onto a sodium dodecyl sulfate-polyacrylamide gel electrophoresis (SDS-PAGE) system, electrophoresed, and transferred to nitrocellulose membranes (Amersham). After blocking with 5% (w/v) nonfat milk in PBS for 2 hours at room temperature, the membranes were incubated with specific antibodies overnight, followed by incubation with horseradish peroxidase-linked secondary antibody (Cell Signaling Technology) for 1 hour at room temperature. The signals were detected by the chemiluminescence phototope-HRP kit (Millipore), according to the manufacturer’s instructions. β-actin was probed as an internal control. All experiments were repeated three times, and similar results were obtained.

### RNA extraction and real-time quantitative RT-PCR

The cells were lysed, and total RNA was isolated using a TRIzol kit (Invitrogen). Then, the RNA was treated with DNase (Promega). Complementary DNA was synthesized according to the manufacturer’s instructions. Real-time quantitative RT-polymerase chain reactions (PCRs) for RARα, retinoic acid receptor beta (RARβ), CCAAT/enhancer binding protein-beta (C/EBP-β), retinoic acid-induced genes E (RIG-E) and interferon regulatory factor 1 (IRF-1), were performed with SYBR Green PCR Master Mixture Reagents (Applied Biosystems) on the Applied Biosystems 7300 real-time RT-PCR system. The specific primers used as follows: 5′-TCTGTGAGAAACGACCGAAAC-3′ and 5′-TGAGGGTGGT GAAGCCG-3′ for RARα gene, 5′-AGTTTGATGGAGTTGGG TGGAC-3′ and 5′-GATGCTGCCATTCGGTTTG-3′ for RARβ, 5′-TCAGCACCC TGCGGAACTT-3′ and 5′-AAGTGCCCCAGTGCCAAAG-3′ for C/EBPβ, 5′-AGG GAGACCGTGTCAGTA GGG-3′ and 5′-CGGAAGTGGCAGAAACCCC-3′ for RIG-E, and 5′-ATGAGACCCTGGCTAGAG-3′ and 5′-AAGCATCCGGTAC ACTCG-3′ for IRF-1. The primers were synthesized by Sangon Biotech (Shanghai, China). All experiments were performed in triplicate. Data were normalized to the housekeeping gene β-actin, and the relative abundance of transcripts was calculated by the comparative ΔΔCT method.

### Redox diagonal electrophoresis

The samples were prepared in 1× SDS sample buffer without any reducing agent and loaded onto 10% SDS-PAGE gels. After the first dimension, non-reducing electrophoresis, the entire lane containing the separated proteins was excised and soaked for 20 min in SDS sample buffer containing 100 mM dithiothreitol to reduce any disulfide bonds present between proteins or within proteins. The gel lane was then rotated 90 degrees and placed horizontally on top of a large-format, 1.5-mm-thick 10% acrylamide gel. Under these conditions, the proteins that do not form disulfide bond electrophorese identically in both dimensions and form a diagonal after the second dimension. In contrast, proteins that contain intra-chain disulfide bond lie above this diagonal, while those that form inter-disulfide bond fall below the diagonal. Finally, immunoblot was performed to identify the dots containing RARα.

### Detection of intracellular ROS level

The cells were incubated with 2′,7′-dichlorodihydrofluorescein diacetate (H_2_DCFDA) (Molecular Probes/Invitrogen) in PBS for 30 min at 37°C while protected from light. The fluorescence intensity, which resulted from the oxidation of the dye, was measured by fluorescence-activated cell sorting (FACS) to determine the level of ROS. The experiments were performed in triplicate.

### Plasmid construction and transfection

Pairs of complementary shRNA oligonucleotides against catalase (5′-AGATGATCTACT CAGAAAT-3′), p65 (5′-GATGAGATCTTCCTACTGT-3′), and non-targeting control NC (5′-TCCCGTGAATTGGAATCCT-3′) were synthesized by Sangon Biotech (Shanghai, China), annealed, and ligated into the pSIREN-RetroQ Vector (Clontech Laboratories) between the *Bam*HI and *Eco*RI sites. A full-length cDNA of human RARα was amplified from NB4 cells by PCR and cloned into the virus expression vector, pMSCV-puro (Clontech Laboratories). shRNA/cDNA-carrying retroviruses were produced in 293 T cells and used to infect NB4 or COS-7 cells. Forty-eight hours after transfection, cells were selected with puromycin (Sigma-Aldrich).

### Immunofluorescence assay

The cells, which were treated as described in the text, were collected onto slides and fixed with 4% paraformaldehyde. After permeabilization with methanol and blocking with 2% (w/v) bovine serum albumin in PBS, the cells were incubated overnight with the antibody against p65. Then, the cells were stained with FITC-labeled anti-rabbit IgG for 1 hour. The cell nuclei were stained with 4′,6-diamidino-2-phenylindole (DAPI, Molecular Probes, Eugene, OR). The stained cells were visualized by fluorescence microscopy (Olympus BX51; Olympus, Tokyo, Japan).

### Patient samples

Patient samples were collected after obtaining informed consent under a procurement protocol that was approved by the Ethics Committee of Rui-Jin Hospital affiliated to Shanghai Jiao Tong University School of Medicine, Shanghai, China. Mononuclear cells were isolated from bone marrow of AML patients using Ficoll-Hypaque liquid (Pharmacia, Piscataway, NJ, USA) and standard procedures.

### Statistical analysis

Results were derived from at least three independent experiments and expressed as the mean ± standard deviation. The Student’s *t*-test was used for statistical analysis. *P* < 0.05 was considered to be statistically significant.

## Results

### Oridonin-induced stabilization of RARα protein in leukemia cells

**Figure 1:**
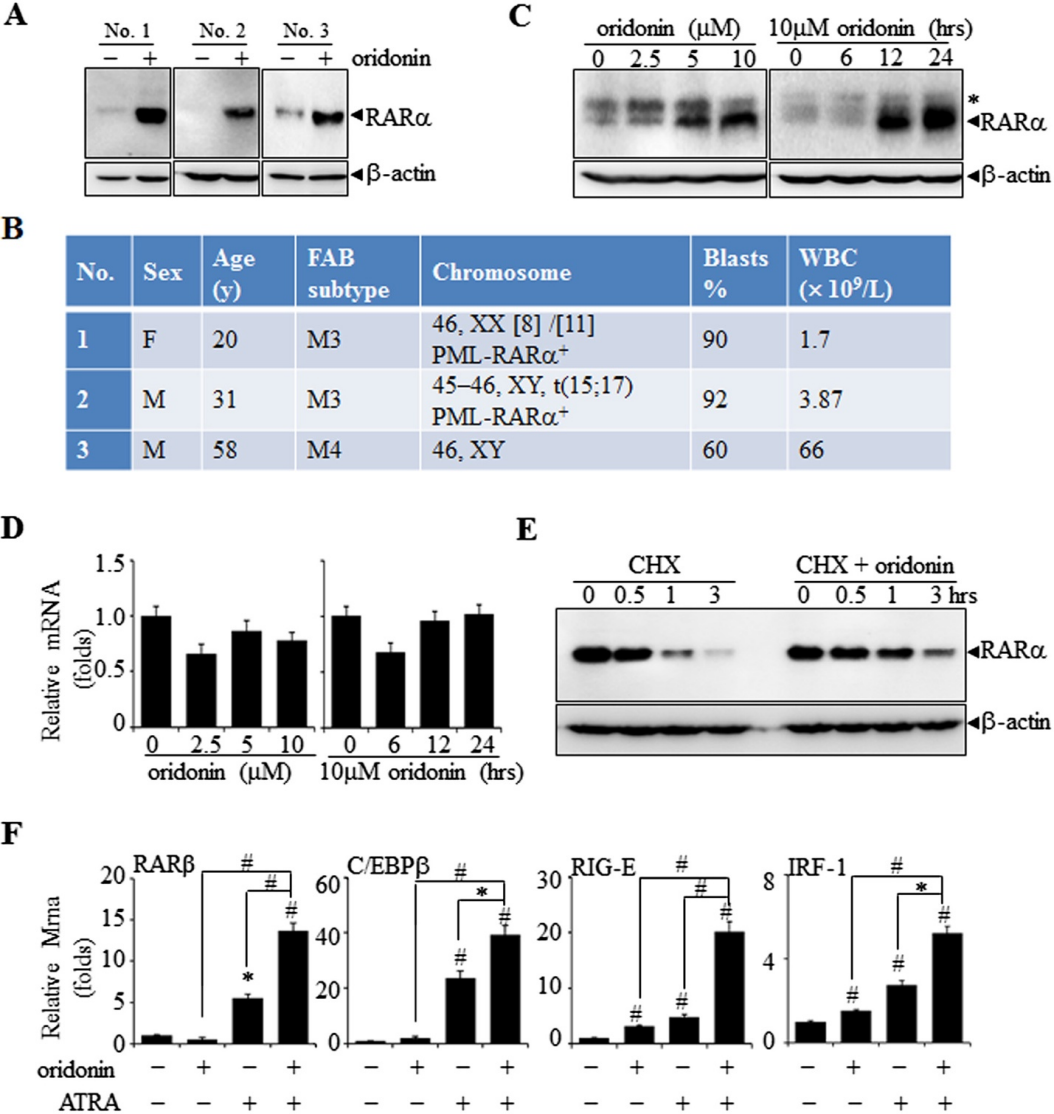
**Oridonin stabilizes RAR**α **protein in leukemia cells. (A)** Primary leukemia cells from three newly diagnosed AML patients were treated with 10 μM oridonin for 12 h, followed by detection of RARα protein with β-actin as a loading control. **(B)** Clinical data of the three AML patients. **(C)** NB4 cells were treated with the indicated concentrations of oridonin for 12 h (left panel) or with 10 μM oridonin for the indicated times (right panel), followed by western blot analysis of the RARα protein with β-actin as a loading control. The symbol * denotes a non-specific protein. **(D)** NB4 cells were treated as described in panel C, followed by the quantification of RARα mRNA by real-time RT-PCR. **(E)** NB4 cells were incubated with 5 μg/mL CHX alone or with 10 μM oridonin for the indicated times. Increased amounts of cell lysates compared with panel A were loaded and then blotted for the RARα protein with β-actin as a loading control. **(F)** NB4 cells were treated with 10 μM oridonin and/or 10 nM ATRA for 48 h, and the mRNA levels of the indicated genes were measured by real-time RT-PCR. The data are represented as fold changes against the control. The symbols * and # represent *P* values less than 0.05 and 0.01, respectively. All experiments were replicated three times and gave consistent results.

Previously, we reported that oridonin increased RARα protein levels and antagonized ATRA-induced RARα loss in leukemia cell lines [23]. To further investigate this, we used oridonin to treat primary leukemia cells and the APL cell line, NB4. The effect of oridonin in increasing the levels of RARα protein could be clearly seen in primary leukemia cells from the bone marrow of three AML patients (Figure 1A). Clinical information of patients is shown in Figure 1B. In NB4 cells, oridonin increased RARα protein levels in a dose-dependent manner (left panel, Figure 1C). When 10 μM of oridonin was applied for 12 h, the levels of RARα protein became significantly increased (right panel, Figure 1C). More interestingly, oridonin failed to modulate the levels of RARα mRNA in NB4 cells (Figure 1D). Moreover, we stably transfected RARα-expressing plasmids into COS-7 cells, and found that oridonin could also increase levels of the ectopically expressed RARα protein (data not shown). These data suggest that oridonin regulates RARα at the post-transcriptional level. In line with this notion, oridonin delayed the degradation of RARα protein in NB4 cells treated with oridonin plus cycloheximide (CHX) compared with cells treated with CHX alone for different times (Figure 1E). We also determined the mRNA levels of four known RARα-targeted genes, RARβ, C/EBP-β, RIG-E, and IRF-1, in NB4 cells with or without oridonin (10 μM) and/or ATRA (10 nM) treatment. Consistent with previous reports [25-28], ATRA treatment alone increased the expression of all four of these genes, and this expression was significantly enhanced by oridonin (Figure 1F). Of note, oridonin alone also slightly, but significantly, increased the expression of RIG-E and IRF-1 but not of RARβ and C/EBPα (Figure 1F). Overall, our results indicate that oridonin can stabilize RARα protein, which shows transcriptional activity in the presence of its ligand, ATRA.

### Involvement of ROS in oridonin-induced RARα stabilization

**Figure 2:**
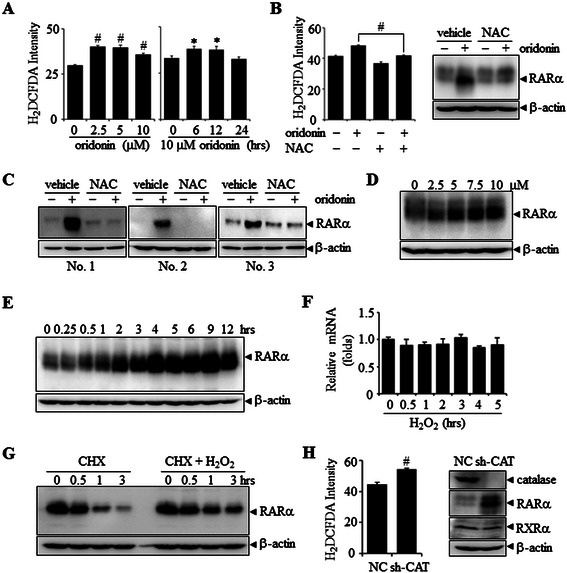
**ROS are involved in oridonin-induced RAR**α **stabilization. (A)** NB4 cells were treated with the indicated concentrations of oridonin for 12 h (left panel) or with 10 μM oridonin for the indicated times (right panel), followed by detection of ROS levels by flow cytometry. The symbols * and # represent *P* values less than 0.05 and 0.01, respectively. **(B)** After pretreatment with or without 2 mM NAC for 1 h, NB4 cells were incubated with 10 μM oridonin for 12 h, followed by detection of ROS levels by flow cytometry (left panel) and western blot detection for RARα protein with β-actin as loading control (right panel). The symbol # represents a *P* value less than 0.01. **(C)** Primary AML cells were treated as NB4 cells in the panel B, and the levels of RARα protein were measured. **(D**, **E)** NB4 cells were treated with the indicated concentrations of H_2_O_2_ for 2 h **(D)** or with 5 μM H_2_O_2_ for the indicated times **(E)**, then the level of RARα protein was assessed. **(F)** NB4 cells were treated with 5 μM H_2_O_2_ for the indicated times, and RARα mRNA levels were evaluated by real-time RT-PCR. **(G)** NB4 cells were incubated with 5 μg/mL CHX alone or in combination with 5 μM H_2_O_2_, followed by western blot detection of RARα protein with β-actin as loading control. **(H)** NB4 cells were infected with pSIREN-RetroQ-derived retroviruses carrying shRNA specifically against catalase (sh-CAT) or non-specific scrambled shRNA as a control (NC). Infected cells were assayed for ROS production (left panel) and western blotted for the indicated proteins. The symbol # represents *P* values less than 0.01, respectively. All experiments were repeated three times and gave consistent results.

Many studies have shown that oridonin can induce oxidative stress [29,30]. Indeed, oridonin rapidly and transiently increased intracellular reactive oxygen species (ROS) levels to a moderate but statistically significant degree in NB4 cells, as assessed by flow cytometric measurement of the ROS probe, H_2_DCFDA (Figure 2A). To investigate whether the increased levels of ROS were involved in oridonin-induced RARα stabilization, we treated NB4 cells with 10 μM oridonin for an additional 12 h after pretreatment with or without the ROS scavenger NAC for 1 h, which totally inhibited oridonin-induced ROS accumulation (left panel, Figure 2B). Of great importance, NAC pretreatment also dramatically abrogated RARα stabilization by oridonin (right panel, Figure 2B). This was also true in primary AML cells (Figure 2C).

We then used H_2_O_2_ to treat NB4 cells to determine the potential role of ROS in RARα stabilization. Intriguingly, direct exposure of a low concentration of H_2_O_2_ obviously increased RARα protein (Figure 2D–E) but not mRNA levels (Figure 2F) in a dose- and time-dependent manner. Furthermore, CHX experiments also demonstrated that H_2_O_2_ delayed the degradation of RARα protein (Figure 2G). In addition, the specific shRNA-mediated knockdown of catalase, a key antioxidant enzyme that eliminates H_2_O_2_ [31], increased endogenous ROS levels in NB4 cells (left panel, Figure 2H). Accordingly, it also increased the abundance of RARα protein (right panel, Figure 2H). Together, these data indicate that a moderately increased level of ROS mediates RARα stabilization.

### Activation of multiple cellular signaling pathways by oridonin

**Figure 3:**
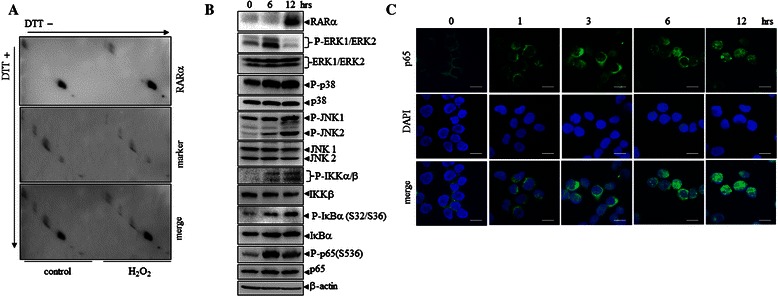
**Oridonin activates multiple cellular signaling pathways. (A)** NB4 cells were treated with 5 μM H_2_O_2_ for 4 h. RARα protein levels were examined by redox diagonal electrophoresis, followed by western blot analysis for RARα. **(B)** NB4 cells were treated with 10 μM oridonin for the indicated times, and cell lysates were western blotted for the proteins indicated. **(C)** NB4 cells were treated with 10 μM oridonin for the indicated times. The intracellular localization of p65 was analyzed by indirect immunofluorescence using anti-p65 antibodies (green). Nuclear DAPI staining (blue) is also shown. Scale bars represent 20 μm. All experiments were repeated three times and gave consistent results.

Next, we addressed how ROS accumulation increases RARα stabilization. We tested whether ROS cause the oxidation of RARα protein by treating NB4 cells with 5 μM of H_2_O_2_ for 4 h, followed by redox diagonal electrophoresis [32]. The results showed that H_2_O_2_ did not directly target RARα protein to cause its oxidative modification (Figure 3A). However, converging lines of evidence indicate that ROS, especially H_2_O_2_, can actually function as signaling messengers and drive several aspects of cellular signaling [33-35]. We showed that oridonin could activate mitogen-activated protein kinases such as ERK1/ERK2 and p38, as well as JNK1 and JNK2, as assessed by their increased phosphorylation (Figure 3B). Of note, levels of phosphorylated ERK1/ERK2 rapidly increased 6 h after oridonin treatment, and then declined after 12 h, indicating that oridonin activates ERK1/ERK2 over a short time. More interestingly, oridonin could also induce phosphorylation of some important components of NF-κB signaling, such as inhibitor kappa B alpha (IκBα) and IKKα/β, indicating that this compound can activate NF-κB signaling (Figure 3B). In addition, oridonin also induced phosphorylation of NF-κB-p65 itself (Figure 3B). Consistently, immunofluorescence staining demonstrated that oridonin treatment induced nuclear localization of NF-κB-p65 (Figure 3C), supporting the idea that oridonin activates NF-κB signaling.

### Suppression of oridonin-induced RARα stability by chemical inhibition of NF-κB signaling

**Figure 4:**
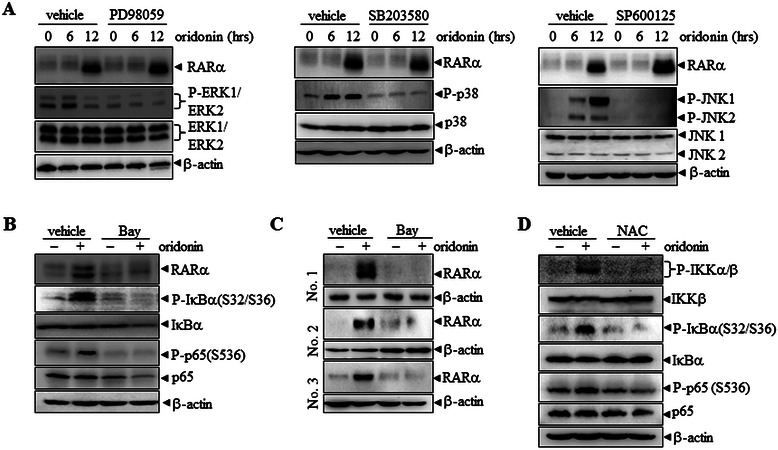
**NF-κB inhibitor blocks oridonin-induced RARαstability.** After pretreatment with and without PD98059, SB203580, SP600125 **(A)**, Bay 11–7082 **(B, C)**, or NAC **(D)** for 1 h, NB4 cells or primary AML cells were treated with 10 μM oridonin for 12 h, followed by western blot analysis of proteins as indicated. All experiments were repeated three times and gave consistent results.

To figure out which pathway(s) mediate oridonin-induced RARα stability, we used specific inhibitors to pretreat NB4 cells for 1 h, followed by oridonin incubation for an additional 12 h. As shown in Figure 4A, pretreatment with PD98059 (ERK inhibitor) or SB203580 (p38 inhibitor) did not influence oridonin-induced RARα stability. In contrast, the JNK inhibitor, SP600125, could slightly enhance oridonin-increased RARα protein levels. The effects of these three kinase inhibitors ruled out the involvement of these pathways in oridonin stabilization of RARα. However, use of the NF-κB signaling inhibitor, Bay 11–7082, significantly inhibited oridonin-induced phosphorylation of IκBα and NF-κB-p65. Interestingly, pre-incubation with Bay 11–7082 antagonized oridonin-increased RARα protein levels in NB4 cells, which indicated that activation of the NF-κB pathway is required for oridonin-induced RARα stability (Figure 4B). Similar results were achieved in AML patient samples (Figure 4C). In addition, NAC preincubation also blocked oridonin-induced phosphorylation of IKKα/β, IκBα and NF-κB-p65 (Figure 4D), consistent with its inhibitory effect on oridonin-stabilized RARα (Figure 2B and C). These data suggested that oridonin stabilized RARα protein via the ROS-activated NF-κB pathway.

### Essential role of activation and nuclear translocation of NF-κB for oridonin-induced RARα stability

**Figure 5:**
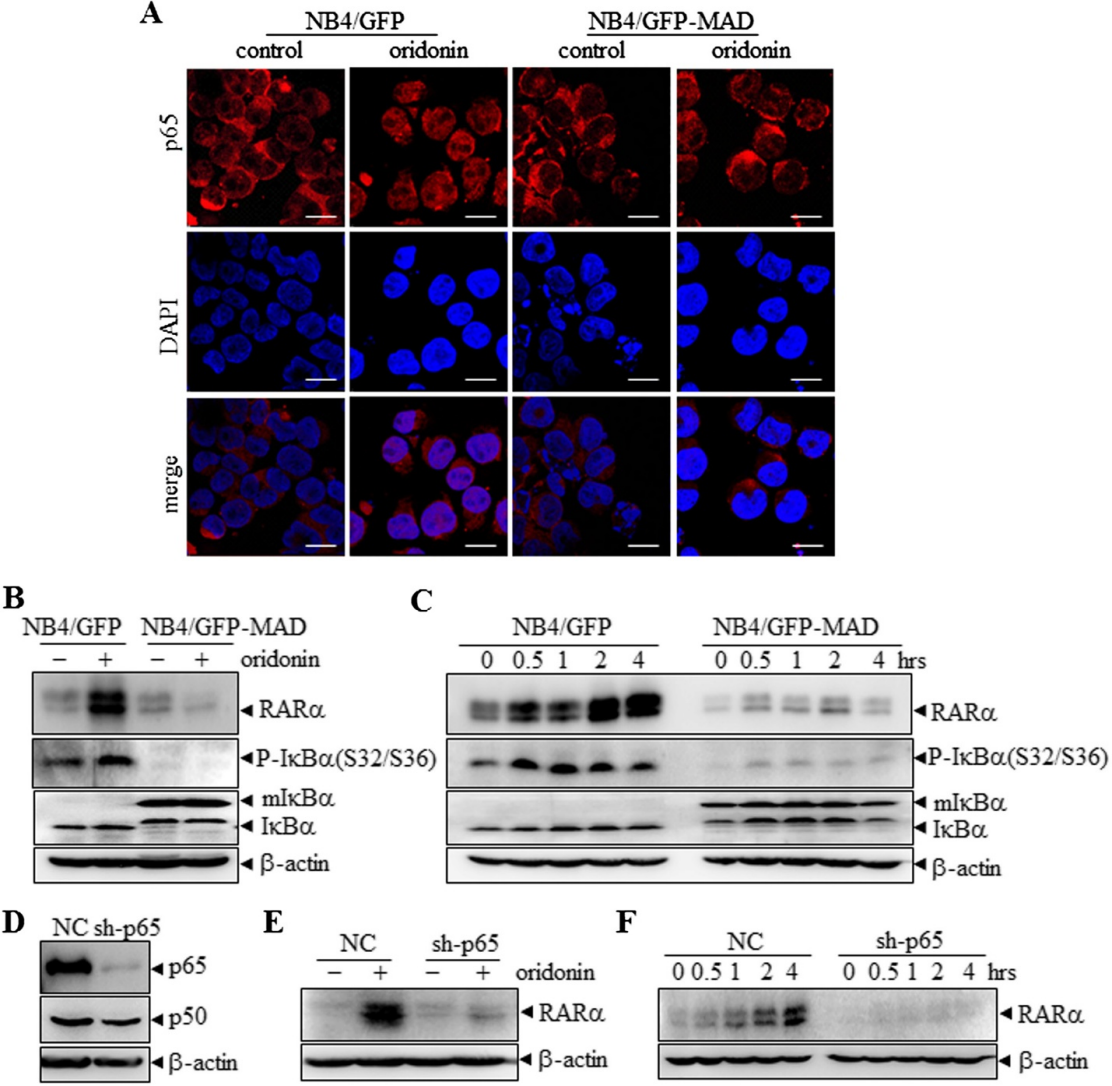
**Oridonin-induced RAR**α **stability requires the activation and nuclear translocation of p65. (A)** NB4/GFP and NB4/GFP-MAD cells were treated with 10 μM oridonin for 12 h. The intracellular localization of p65 was analyzed using anti-p65 antibodies (red) with DAPI staining (blue) for nuclei. Scale bars represent 20 μm. **(B, C)** NB4/GFP and NB4/GFP-MAD cells were treated with 10 μM oridonin for 12 h **(B)** or treated with 5 μM H_2_O_2_ for the times indicated **(C)**, and the cell lysates were western blotted for the indicated proteins. **(D)** NB4 cells were infected with pSIREN-RetroQ-derived retroviruses carrying shRNA for p65 or scrambled shRNA as a control, and the cell lysates were western blotted for the indicated proteins. **(E, F)** NB4-NC and NB4-sh-p65 cells were treated with 10 μM oridonin for 12 h **(E)** or with 5 μM H_2_O_2_ for the times indicated **(F)**, and the cell lysates were western blotted for proteins as indicated. All experiments were repeated three times and gave consistent results.

To confirm that oridonin stabilizes RARα through the NF-κB pathway, we used NB4/GFP-MAD cells to perform further experiments. This engineered cell line stably expresses the GFP-tagged super-repressor form of IκΒα, namely IκΒα (A32/36), which confers cellular resistance to signal-induced phosphorylation and subsequent proteasome-mediated degradation of IκΒα, resulting in the constitutive suppression of NF-κB activation by sequestering it in the cytoplasm [36]. As shown in Figure 5A, the over-expression of IκΒα (A32/36) blocked oridonin-induced nuclear translocation of p65. As expected, both oridonin- and H_2_O_2_-induced RARα stability were inhibited in NB4/GFP-MAD cells compared with NB4/GFP cells (Figure 5B and C). Furthermore, we stably transfected NB4 cells with shRNA specifically against the p65 subunit of the NF-κB family, which effectively silenced the expression of p65 but not p50 (Figure 5D). Notably, p65 knockdown prevented oridonin and H_2_O_2_-induced RARα stability in NB4 cells (Figure 5E and F). Collectively, these results suggest that the activation and nuclear translocation of p65 is essential for oridonin to stabilize RARα.

### Promotion of p65 nuclear translocation increases RARα stability

**Figure 6:**
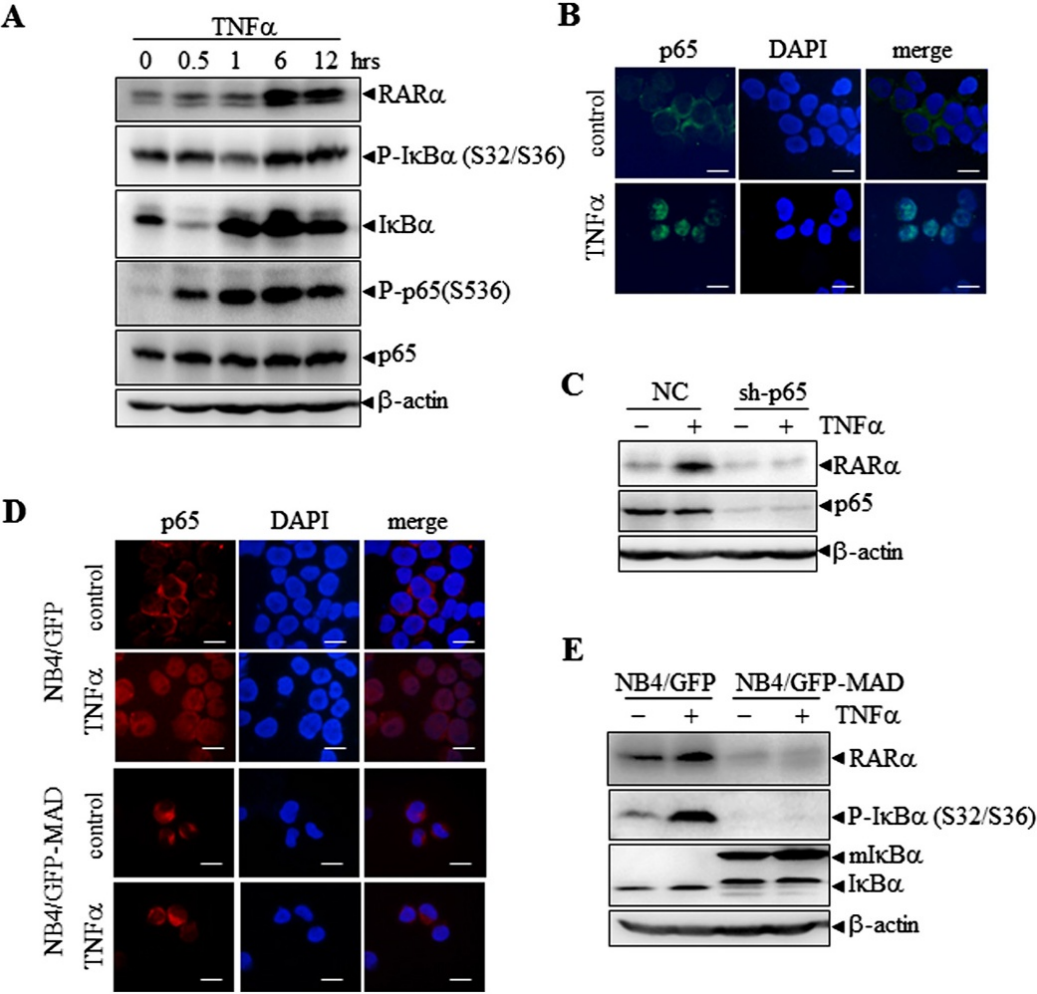
**TNFα stabilizes RAR**α **protein by activating NF-κB. (A)** NB4 cells were treated with 10 ng/mL TNFα for the times indicated, followed by western blotting for proteins as indicated. **(B)** NB4 cells were treated with 10 ng/mL TNFα for 0.5 h, followed by immunofluorescent staining using anti-p65 antibodies. **(C)** NB4 cells with NC or sh-p65 infection were treated with 10 ng/mL TNFα for 12 h, followed by western blot analysis for proteins as indicated. **(D)** NB4/GFP and NB4/GFP-MAD cells were treated with 10 ng/mL TNFα for 0.5 h, and then the intracellular localization of p65 was analyzed by immunofluorescence. **(E)** NB4/GFP and NB4/GFP-MAD cells were treated with 10 ng/mL TNFα for 12 h. The cell lysates were western blotted for the indicated proteins. All experiments were repeated three times and gave consistent results.

It is well known that TNFα is a classical activator of NF-κB signaling; therefore, we investigated the consequence of TNFα treatment on RARα expression to address whether oridonin-induced RARα stability is mediated specifically by ROS-activated NF-κB activation. Our results showed that TNFα treatment also resulted in a strong increase in RARα expression together with activation and nuclear translocation of NF-κB-p65 in NB4 cells (Figure 6A and B). This TNFα-induced RARα stability could be inhibited by p65 knockdown (Figure 6C). In addition, the over-expression of IκΒα (A32/36) blocked the nuclear translocation of p65 and RARα stability induced by TNFα (Figure 6D and E). All these data support the idea that translocation of p65 nuclear induces RARα stability.

## Discussion

In this study, we report that the natural diterpenoid, oridonin, induces a moderate production of cellular ROS that activates upstream of the NF-κB signaling pathway to cause nuclear translocation of p65, which is responsible for oridonin-stabilized RARα protein. These findings indicate that moderate oxidative stress induced by oridonin may change the intrinsic mechanisms that regulate RARα protein stability through the NF-κB signaling pathway, which provides a new perspective of oridonin as a candidate anti-neoplastic drug.

The modulation of RARα by ATRA during APL treatment has stimulated considerable interest in RARα metabolism and its potential therapeutic mechanism [37]. ATRA activates RARα signaling with subsequent effects on differentiation, while at the same time steady-state RARα protein levels are markedly reduced [12]. RARα, as the receptor for ATRA, is required for its action; therefore, RARα degradation is thought to be an inbuilt resetting mechanism to make ATRA signaling self-limiting. Therefore, it is possible that stabilizing the RARα protein can optimize this signaling, which indicates that RARα could be a potential target for cancer therapeutics. Recently, several studies have demonstrated that some compounds, such as lithium chloride (LiCl) [38], granulocyte-colony stimulating factor [38], STI571 [39], di-*tert*-butyl-benzohydroquinone [40], Pharicin B [15], and oridonin [23], which are capable of attenuating ATRA-induced loss of RARα protein, have been shown to enhance ATRA-induced differentiation. However, the underlying mechanism of RARα accumulation has not been fully described. In this work, we used oridonin as a probe to show that a moderate level of oxidative stress can stabilize RARα protein through the nuclear translocation of p65. Further investigation is needed to test whether this mechanism can be extended to other small molecules with similar RARα-stabilizing ability. In addition, because RARα is an essential transcriptional and homeostatic regulator of a plethora of physiological processes, numerous investigations have established correlations between down-regulation of RARα and malignant progression. In addition to APL, this has been observed in cervical carcinoma [41], skin tumors [42], motor neuron disease [43], and breast cancer [44]. In this context, stabilizing RARα may permit optimized use of retinoids in cancer prevention and treatment, which warrants further investigation.

It is now widely accepted that a moderate degree of ROS can play an important role in determining cell fate through the modulation of cellular signaling and gene expression [45,46]. For example, elevated but sub-lethal levels of ROS can modulate the differentiation of various types of cells, such as hematopoietic cells [47,48], neurons [49], embryonic stem cells [50], osteoclasts [51], and cardiac stem cells [52]. However, little is known regarding the molecular targets of ROS. Here, we found that moderately increased levels of ROS are crucial for oridonin-induced RARα stabilization, which may account for the anti-neoplastic mechanism of oridonin. It is tempting to suggest that this newly identified mechanism may underlie similar differentiation effects of some natural diterpenoids. Nevertheless, attention should be paid to the cell type, as well as to the extent and duration of ROS increase, as these factors can determine the precise consequences of the cellular response to oxidative stress. For instance, a relatively high concentration of H_2_O_2_ (0.1 mM) can suppress retinoid signaling through the proteasomal degradation of RARα [14].

The NF-κB family is a group of transcriptional factors consisting of p65 (RelA), RelB, c-Rel, p50/p105, and p52/p100. In the classical NF-κB signaling pathway, the p50/p65 dimer is sequestered in the cytoplasm by IκΒα. After stimulation, IκΒα is phosphorylated and consequently degraded through the proteasomal pathway. Thus, the p50/p65 dimer is released, translocates to the nucleus, and activates target genes [53]. In this report, we revealed that oridonin stabilizes RARα protein by inducing nuclear translocation of p65, which was evidenced by the use of the ROS scavenger, NAC, the NF-κB inhibitor, Bay 11–7082, IκΒα (A32/36) over-expression, and p65 knockdown. Moreover, we tested whether TNFα, a classical activator of NF-κB signaling, modulates stability of RARα protein. As expected, TNFα treatment also strongly increased RARα expression, which may account, at least in part, for TNFα-induced differentiation in some leukemia cells [54,55]. Previous studies indicated that oridonin mainly activates the upstream of the NF-κB signaling pathway, while its inhibitory effect is due to the direct interference of NF-κB DNA binding activity [56-59]. Leung et al. demonstrated that oridonin decreased the DNA binding activity of NF-κB without interfering with p65 translocation [59]. Of note, the exact mechanisms by which activated NF-κB stabilizes RARα protein require further investigation.

## Conclusions

Our results indicate that oridonin stabilizes RARα protein by increasing the levels of cellular ROS, followed by activation of the NF-κB signaling pathway. Accordingly, the NF-κB activator, TNFα, can also increase the stability of RARα protein. These findings suggest a new mechanism underlying the regulation of RARα protein stability and shed new light on understanding potential therapeutic roles of oridonin in leukemia and other RARα-related diseases.

## Abbreviations

AML: Acute myeloid leukemia
APL: Acute promyelocytic leukemia
ATRA: All-*trans* retinoic acid
CHX: cycloheximide
H_2_O_2_: Hydrogen peroxide
IκB: Inhibitor of NF-κB
IKK: IκB kinase
LiCl: Lithium chloride
NAC: *N*-acetyl-l-cysteine
NF-κB: Nuclear factor-kappa B
PML: Promyelocytic leukemia
RARα: Retinoic acid receptor alpha
ROS: Reactive oxygen species
RXRs: Retinoid X receptors
shRNAs: Short hairpin interfering RNAs
